# Costs and effects of comprehensive geriatric assessment in primary care for older adults with high risk for hospitalisation

**DOI:** 10.1186/s12877-021-02166-1

**Published:** 2021-04-21

**Authors:** Magnus Nord, Johan Lyth, Jenny Alwin, Jan Marcusson

**Affiliations:** 1 Primary Health Care Center Valla, and Department of Health, Medicine and Caring Sciences, Linköping University, Linköping, Sweden; 2 Department of Health, Medicine and Caring Sciences, Linköping University, Linköping, Sweden; 3 Department of Acute Internal Medicine and Geriatrics, Linköping University, Linköping, Sweden

## Abstract

**Background:**

The healthcare system needs effective strategies to identify the most vulnerable group of older patients, assess their needs and plan their care proactively. To evaluate the effectiveness of comprehensive geriatric assessment (CGA) of older adults with a high risk of hospitalisation we conducted a prospective, pragmatic, matched-control multicentre trial at 19 primary care practices in Sweden.

**Methods:**

We identified 1604 individuals aged 75 years and older using a new, validated algorithm that calculates a risk score for hospitalisation from electronic medical records. After a nine-month run-in period for CGA in the intervention group, 74% of the available 646 participants had accepted and received CGA, and 662 participants remained in the control group. Participants at intervention practices were invited to CGA performed by a nurse together with a physician. The CGA was adapted to the primary care context. The participants thereafter received actions according to individual needs during a two-year follow-up period. Participants at control practices received care as usual. The primary outcome was hospital care days. Secondary outcomes were number of hospital care episodes, number of outpatient visits, health care costs and mortality. Outcomes were analysed according to intention to treat and adjusted for age, gender and risk score. We used generalised linear mixed models to compare the intervention group and control group regarding all outcomes.

**Results:**

Mean age was 83.2 years, 51% of the 1308 participants were female. Relative risk reduction for hospital care days was − 22% (− 35% to − 4%, *p* = 0.02) during the two-year follow-up. Relative risk reduction for hospital care episodes was − 17% (− 30% to − 2%, *p* = 0.03). There were no significant differences in outpatient visits or mortality.

Health care costs were significantly lower in the intervention group, adjusted mean difference was € − 4324 (€ − 7962 to − 686, *p* = 0.02).

**Conclusions and relevance:**

Our findings indicate that CGA in primary care can reduce the need for hospital care days in a high-risk population of older adults. This could be of great importance in order to manage increasing prevalence of frailty and multimorbidity.

**Trial registration:**

clinicaltrials.gov Identifier: NCT03180606, first posted 08/06/2017.

## Background

Increasing health care needs among older adults are recognised as a major challenge to the healthcare system in developed countries [1, 2]. The majority of older adults is healthy, but frailty and multimorbidity increase with age. Frailty can be described as a state of increased vulnerability of the old person with increased risk of adverse outcomes [3], but there is a lack of consensus on its definition [4] and there are several different ways to measure frailty in research and clinical practice [5]. In this study we use the cumulative deficit model to assess frailty [6]. The prevalence of frailty in community-dwelling older adults is estimated to be 20–40% at the age of 75 years [7]. Frail older adults are high users of medical services and recent studies have demonstrated a two-to-three times higher incidence of unplanned hospital admissions among frail individuals compared with non-frail people [8, 9]. Numerous intervention-studies have been performed around the world during the last decades to identify effective treatments and strategies to manage increasing healthcare needs among older persons [10, 11].

Comprehensive geriatric assessment (CGA) is accepted as the gold standard for the management of frailty [12, 13]. CGA is described “a multidimensional, multidisciplinary process which identifies medical, social and functional needs and the development of an integrated/co-ordinated care plan to meet those needs” [14]. The positive effects of CGA include slowing down functional decline and reduction of hospitalisations and admissions to nursing homes [15, 16]. CGA has been modified and adapted to various settings; a wide range of CGAs using different instruments with different intensity has been studied in primary care [14, 16–18]. In spite of a great number of intervention studies, there is insufficient evidence for effectiveness of CGA in primary care regarding reduced mortality or inpatient care [11, 17]. There is also limited economic evaluation that suggests that CGA may save on hospital costs [19, 20]. One of the highlighted challenges in these studies is the identification of older adults in primary care that would benefit most from CGA interventions [17]. Recently, studies using electronic administrative and medical record health care data to detect frailty and predict risk for hospitalisation have provided new tools for this. Using algorithms is a way to detect and grade frailty that does not require manual and time-consuming contacts with patients [21–23]. A high electronic frailty index corresponds to high risk for hospitalisation [22].

Therefore, we decided to use an algorithm for prediction of hospitalisations to identify a target group for CGA [23]. As previous studies have identified difficulties in implementing complex interventions in primary care [24], we designed a CGA intervention that was person-centred and differentiated according to individual needs. We used a CGA tool with a limited set of items performed by a small nurse-physician team to adapt it to the primary care context [25].

The aim was to examine if comprehensive geriatric assessment adapted to primary care and delivered to a risk group identified by a prediction model can reduce unplanned hospitalisations.

## Methods

We conducted a prospective multicentre trial at 19 primary care practices in the county of Östergötland in Sweden as previously described [26].

We rated the trial design as pragmatic according to all ten domains of the PRECIS-tool (Pragmatic Explanatory Continuum Indicator Summary) [27] as we studied the effects of implementation of a new work-mode without extra measurements or assessments in the intervention or control groups. As this work-mode was already decided by the county, participants were not asked for consent, in accordance with the decision of the ethical board.

We decided to start the collection of healthcare data (the follow-up period) when 90% of all available participants in the intervention group that accepted to participate had received the CGA (the run-in period). Our original plan was a run-in period of 3 months, but we had to prolong this period to 9 months because the start-up process required more time than expected. The staff information and training started in January 2017, recruitment of patients for CGA in April 2017 and the follow-up period of 24 months started in January 2018.

### Setting

We were unable to randomise the practices, therefore we decided to compare the volunteering intervention practices with care-as-usual at matched practices in the county. We identified practices with similar locations, size and populations over 75 years of age and selected them as controls.

The practices were situated in both rural and densely populated areas with listed populations from 6000 to 21,000 inhabitants. Together, the 19 practices covered 40% of the total population aged 75 years and older across the county. Prior to the intervention, there was no tradition of performing CGA in primary care. Primary care in Sweden is financed by the county council and organised in primary care practices with a typical population of 6000–20,000 persons and an average workforce of 3–10 physicians, 6–15 nurses and administrative staff. Nurses are an important part of the workforce and have experience of working independently with both acute illness and chronic diseases, which makes them capable of performing a significant part of the CGA. The intervention practices did not receive any extra staff linked to the intervention.

### Staff training and monitoring

The practices in the intervention received an introductory visit of 2 h during the run-in period where researchers introduced the CGA tool, the concept of frailty and the other features of the intervention to the nurses and physicians involved. During the whole study period, we visited the practices every 6 months giving advice and answering questions. Between these visits, the nurses at the intervention practices had access to a supervisor via email or telephone for additional support. In addition, we organised network meetings every 6 months where nurses and physicians at the intervention practices shared experiences, discussed CGA and related topics. There was no interaction with the control practices.

### Participants

We identified 1604 individuals aged 75 years or older at the participating practices in March 2017. We used a recently developed and validated prediction model that contains 38 variables identified with multivariable logistic regression [23]. Age and healthcare use are the principal predictors, together with diagnoses from inpatient care and outpatient visits. Data from the preceding 12 months of electronic medical records was extracted to calculate a risk score for unplanned hospital admission. Our participants constituted the 11% with the highest risk score of the aged population. There were no exclusion criteria; all the selected individuals were included. We distributed a list of participants to the intervention practices at the end of March 2017.

### Sample size

We hypothesised a reduction in hospital care days of 20% as a result of the intervention based on an earlier study where a reduction was found for individuals at high risk [28]. In a pilot study, we found an incidence of hospitalisations over 60% in the predicted target population. A calculation based on this, a power of 0.8 and a significance level of 0.05 led to a minimum number of participants of 270 in each group. We estimated a dropout rate of 30% giving a number of 380 participants in each group. Then we doubled the number of participants taking into account that the heterogeneity of the practices and the participants would lower the likelihood of detecting a meaningful intervention effect.

### Intervention

The intervention comprised two main components. The first component involved presentation of the list of participants to the intervention practices. The list included the risk score and the number of hospitalizations, visits to emergency room and to any physician during the preceding 12 months for each participant. In this way, the practices were made aware of a group of older adults at high risk for hospitalisation.

The second component was the comprehensive geriatric assessment (CGA) performed by nurses at the intervention practices together with the listed/responsible physician of the participant. The CGA was carried out during the run-in period between April and December 2017. The primary care nurses contacted the participants in the intervention group by telephone and offered them a health evaluation/CGA and follow-up at the practice. Around 10% of the participants had greater reduction of mobility and received the CGA at home. We constructed an instrument for the CGA in this intervention, the Primary care Assessment Tool for Elderly (PASTEL) [25]. This 4-page form contains two parts. The first part is performed by a nurse and contains self-rating of health and about 20 items covering different perspectives of health and frailty including for example social network, vision, hearing, falls, incontinence, weight loss and psychological problems. This is followed by a medication review and physical measures. It ends with questions to the patient regarding the main concerns about their health and their needs in the future.

The second part is a template for a team meeting where the responsible physician and the nurse that performed the initial assessment together grade frailty with Clinical frailty scale [6] and review a check-list for further investigation and supportive actions, based on the assessment, medical records and personal knowledge. Typically, the assessment by the nurse lasted 1 h and the team meeting 15–30 min. In some cases, the participant was not known by the physician, and a visit for further medical assessment was planned. The participants did not participate in the team meeting, but was contacted by the nurse and involved in the care planning afterwards.

After the CGA, the participants were given care according to their individual needs. This included referral to occupational therapy, physiotherapy or other specialist services when needed. We encouraged the teams to provide continuity for the participants to both physician and nurse and to facilitate accessibility to the practices for the participants. The intervention had no standard treatment or action that was offered to all participants except the CGA described above. We instructed the teams to follow their clinical judgement and to individualise treatment and follow-up intervals. Control practices performed care as usual. We did not gather information about what specific actions the participants received.

### Outcomes

Primary outcome was number of hospital care days.

Secondary outcomes were number of hospital care episodes and number of outpatient visits, health care costs and mortality. In analyses, outpatient visits were also subdivided into visits in emergency care, primary care and other outpatient care.

Using the unique 10-digit Swedish personal identity numbers, we linked patient data to the Care Data Warehouse in Östergötland (CDW) and Cost Per Patient database (CPP). The data warehouse includes all healthcare contacts (inpatient, outpatient and primary care) for both private care and public care, and the cost database includes costs for all contacts within public care. For an adequate assessment of cost data, all costs collected from CPP were adjusted to the price level of 2019 with an increase of historical costs with 3% per year. We converted costs to euros (one € = 10 SEK). Costs were missing in 11% of all contacts, mainly because of care given by private primary care providers. For these contacts, healthcare costs were imputed with the average cost calculated per contact type for existing data. Contact types were inpatient care, physician visit, and visit with a professional other than a physician. Less than 0.03% of contacts with missing costs were related to inpatient care. We also collected the number of co-morbidities from CDW as defined by the Royal College of Surgeons Charlson Score [29].

### Statistical analysis

We compared baseline characteristics; risk score, age and gender, between the intervention group and the control group. All outcomes were analysed according to intention to treat. Continuous data were analysed with t-test and categorical data were analysed with χ2-test. We used generalised linear mixed models to compare the intervention group and control group regarding all outcomes, controlling for baseline covariates. We expected that two patients in the same primary care centre were likely to have more similar rates of the different outcomes than two patients in randomly different primary care centres. Therefore, our models included primary care centre as a random intercept effect. Risk score, age and gender were used as fixed covariates. For healthcare use data, we selected the distribution for our regression models based on an assessment of Pearson residuals. Since we found evidence of over-dispersion in healthcare use data, we preferred a negative binomial model with a log-link over a Poisson model. For cost data, we used a normal distribution with an identity link.

For healthcare use data, we calculated event rates for the two groups from actual data. From these event rates, absolute risk reductions for the intervention group were calculated. The relative risk reductions (RRRs) for the intervention group were analysed by the multivariable mixed count data models. For cost data, mean costs and mean differences between the groups were calculated from actual data. Adjusted mean differences were analysed by the multivariable mixed cost models.

We analysed mortality (1-overall survival) by the Kaplan-Meier method with any cause of death as an event and patients were censored by the end of follow-up 31 December 2019. Hazard ratio (HR) with 95% CIs between the intervention group and control group were estimated by multivariable Cox regression. The Cox regression was adjusted for the baseline covariates, risk score, age and gender.

For the primary outcome, the level of significance was set at 0.05 (two-tailed). Despite multiple secondary outcomes, we decided to keep the level of significance of 0.05 in order to detect meaningful differences. Therefore, these analyses should be interpreted with care. All analyses, except for Kaplan-Meier, were performed using SPSS version 25.0 (IBM Corp., Armonk, NY). The Kaplan-Meier plot was produced in R version 3.5.2 (R Core Team, Vienna, Austria).

## Results

**Fig. 1:**
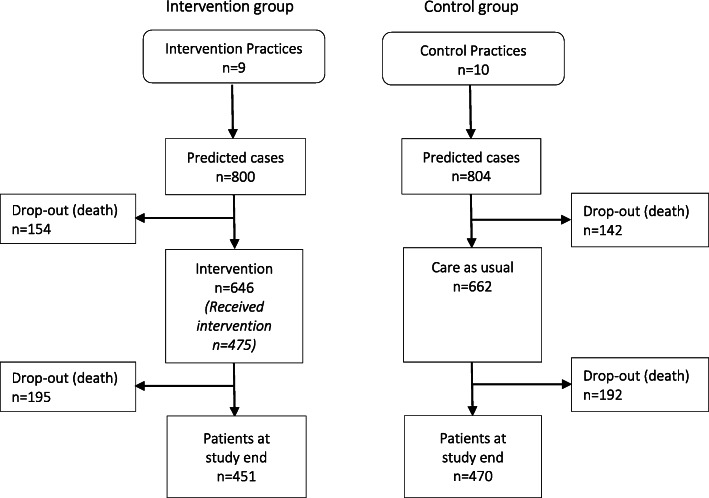
Flow of participants

The 1604 participants were equally distributed between intervention and control practices. During the run-in period (1 April 2017–31 December 2017) there were 17% (control) and 19% (intervention) dropouts caused by death. Among the remaining 1308 participants, mean age was 83.2 years and 51% were female. In the intervention group, 475 of the 646 participants (74%) accepted the invitation to the comprehensive geriatric assessment (CGA) and received the intervention (Fig. 1).

Table 1 shows the characteristics of the participants. There were no significant differences regarding age, gender, risk score or comorbidities between the intervention and control groups.

**Table 1:** Baseline characteristics comparing intervention group and control group

Measure	Intervention group (***n*** = 646)	Control group (***n*** = 662)
Age, mean (SD)	83.0 (5.5)	83.3 (5.5)
Gender, number (%)		
Men	325 (50.3%)	315 (47.6%)
Women	321 (49.7%)	347 (52.4%)
Risk score, mean (SD)	0.35 (0.18)	0.33 (0.17)
Number of co-morbidities		
0	48 (7.4%)	47 (7.1%)
1	112 (17.3%)	95 (14.4%)
2	143 (22.1%)	126 (19.0%)
3 or more	343 (53.1%)	394 (59.5%)

### Primary outcome

We found a significant reduction in hospital care days in the intervention group (8.5 days vs 10.3 days) during the 2 years of follow-up. Relative risk reduction (RRR) was −22% (−35% to −4%), *p* = 0.02 (Table 2).

**Table 2:** Results for primary and secondary outcomes of healthcare use comparing intervention group and control group from 1 Jan 2018 until 31 Dec 2019

Outcomes	Group	No. of events/No. of participants	Event rates	Absolute risk-reduction	Relative risk-reduction (95% CI)^**a**^	***P*** value
Total number of hospital care days	Intervention	5500/646	8.5	−1.8	−22% (−35% to − 4%)	**0.02**
	Control	6833/662	10.3			
-No. of hospital care episodes	Intervention	922/646	1.4	−0.3	−17% (−30% to −2%)	**0.03**
	Control	1109/662	1.7			
Total Number of visits	Intervention	28,325/646	43.8	−0.7	−4% (−15 to 8%)	0.50
	Control	29,471/662	44.5			
-No. of primary care visits	Intervention	16,500/646	25.5	0.9	0% (−20 to 26%)	0.99
	Control	16,300/662	24.6			
-No. of emergency room visits	Intervention	1512/646	2.3	−0.3	−10% (−23 to 5%)	0.20
	Control	1718/662	2.6			
-No. of other outpatient care visits	Intervention	10,315/646	16.0	−1.3	−10% (−25 to 8%)	0.25
	Control	11,444/662	17.3			

^a^Relative risk reductions were analysed with mixed models using primary care centres as random intercept. All models were estimated by a negative binomial distribution with a log link and were adjusted for risk score, age and gender. *CI* Confidence interval. Significant results are marked with bold text

### Secondary outcomes

**Fig. 2:**
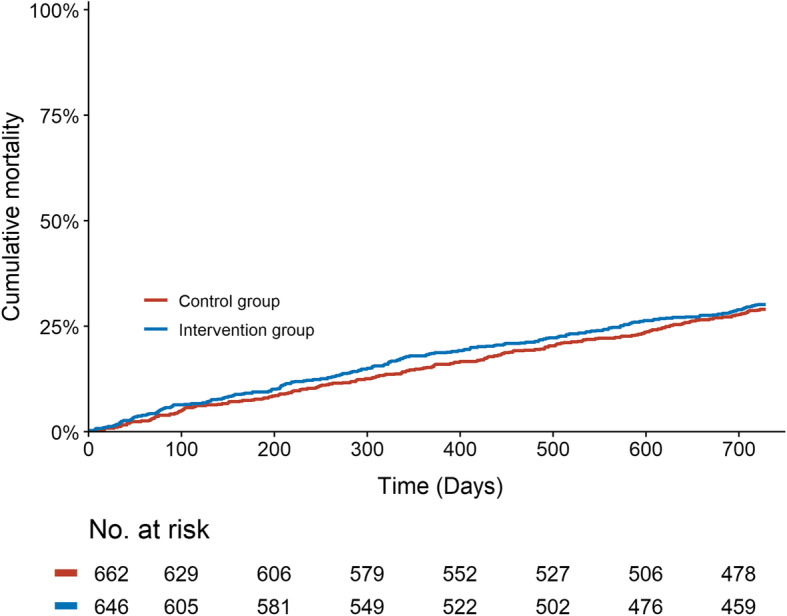
Mortality comparing intervention group (*n* = 646) and controls (*n* = 662) from 1 Jan 2018 until 31 Dec 2019

Relative risk reduction for hospital care episodes was − 17% (− 30% to − 2%) *p* = 0.03. The number of outpatient visits to primary or secondary care did not differ significantly (Table 2). Mortality was similar in the two groups (Fig. 2), the adjusted Cox regression resulted in a HR of 1.1 (95% CI: 0.9 to 1.3, *p* = 0.56).

During the 2 years of follow-up, the average healthcare costs in the intervention group and the control group were **€** 22,250 and **€** 25,245 respectively (Table 3). When we adjusted for age, gender and risk score we found a significantly lower total cost in the intervention group compared to the control group (**€** - 4324, 95% CI: **€** - 7962 to - 686, *p* = 0.02). This corresponds to a 17% lower cost in the intervention group. A lower cost for hospital care episodes (**€** − 2994, 95% CI: **€** -5690 to − 297, *p* = 0.03) in the intervention group compared to the control group was the main contributing factor for the lower total healthcare cost.

**Table 3:** Results for healthcare costs (€) comparing intervention group (*n* = 646) and control group (*n* = 662) from 1 Jan 2018 until 31 Dec 2019

Outcomes	Group	Unadjusted mean cost (€)	Unadjusted mean difference (€)	Adjusted mean difference € (95% CI)^**a**^	***P*** value
Total cost of hospital care episodes	Intervention	10,810	-2165	−2994 (− 5690 to − 297)	**0.03**
	Control	12,975			
Total cost of visits	Intervention	11,440	− 830	− 1369 (− 2923 to 186)	0.08
	Control	12,270			
-Cost of primary care visits	Intervention	4009	− 326	−297 (− 909 to 314)	0.34
	Control	4335			
-Cost of emergency room visits	Intervention	1253	− 131	− 170 (− 378 to 38)	0.11
	Control	1384			
-Cost of other outpatient care visits	Intervention	6178	− 373	− 855 (− 2205 to 495)	0.21
	Control	6551			
Total cost of visits and hospital care episodes	Intervention	22,250	− 2995	− 4324 (−7962 to −686)	**0.02**
	Control	25,245			

^a^Adjusted mean differences were analysed with mixed models using primary care centres as random intercept. All models were estimated by a normal distribution with a identity link and were adjusted for risk score, age and gender. *CI* Confidence interval. Significant results are marked with bold text

## Discussion

This study combined CGA with digital prediction in primary care covering 19 practices and 1604 adults over 75 years of age. The intervention significantly reduced the risk for prolonged hospital stay, expressed as number of hospital care days (relative risk reduction RRR − 22%) in a high-risk population of older adults.

Our secondary outcome data indicated a decreased risk for hospital admission (RRR − 17%), without any significant risk difference in mortality or in the number of outpatient visits, including primary care visits. Altogether, this corresponded to a healthcare cost reduction of **€** 4324 (− 17%) for each patient in the intervention group during the 2 years of follow-up compared to usual care.

The principal strength of this trial is the pragmatic design of the intervention, allowing us to study the effects in a context very close to everyday practice. The design also allowed us to compare our intervention with usual care that was not affected by repeated visits and measurements of the controls, which could be confounders and mask the true effect [30]. Secondly, we did not exclude any of the individuals that we identified with the prediction model and we collected data relating to every participant from the healthcare database with very little missing data. Thirdly, we used a CGA tool with a limited set of items and scales and freedom for the staff to tailor further assessments and actions to the individual. We believe that this facilitates implementation and makes the work-mode adapted to the primary care context.

The trial has several limitations: Firstly, we could not randomise the participating practices. There may be differences between the practices that we are not aware of and that could influence the results. The mixed method analysis using primary care centres as the random intercepts adjusted the results to some extent for such differences.

Secondly, we do not have data on what actions were given to each participant after the CGA, thus we cannot tell if the effect was conferred by the CGA itself or by the subsequent actions or referrals. This would have been valuable but our study design where we did not approach controls or participants that did not get the CGA did not allow us to collect these data. Furthermore, the statistical power of the study was not enough to detect differences related to the actions or referrals.

Thirdly, a longer run-in period than planned made the start of the intervention more outstretched. That may also have excluded a proportion of more frail individuals from participating, which possibly reduced the effect of the intervention.

### Comparison with other studies

Our results support the hypothesis that CGA is effective in reducing healthcare needs in older adults. The size of the effect is comparable with other interventions that reduced hospitalisations, both randomised [28, 31, 32] and non-randomised. A number of other studies have failed to demonstrate reduction in hospitalisations or admission to nursing homes [33–37]. One of the suggested explanations for this is the difficulty in recruiting and following participants who are frail enough [38]. Our population had a substantially higher mean hospitalisation incidence and mortality than most comparable studies [28, 32–34, 37]. Other possible explanations for failure in other studies to observe significant effects include small samples and short follow-up times. Our design allowed us to access a big, high-risk sample that we could follow for 24 months and collect data from all participants. The prediction model that we used to select participants is based on healthcare needs, age and selected diagnoses [23]. The high mortality, co-morbidities and rate of hospitalisation among our participants support that this model concords with electronic frailty indexes [22] and other frailty measures [6].

There is a range of studied CGA interventions, from an intense geriatrician-led ambulatory unit intervention [32, 33, 39] to a more limited CGA with a team consisting of GP, a nurse and a social worker [15, 20, 40]. We decided to use a doctor-nurse team and a CGA tool with a relatively small set of items aiming for a differentiated and individualized intervention [25]. A more complex and intensive intervention would restrict the intervention to a smaller group and probably make it less feasible for broad implementation.

## Conclusions

In this study, we have demonstrated that CGA can be performed in a primary care context and significantly reduce the need for hospital care. Furthermore, the prediction model succeeded to identify a target group that could benefit from this design of CGA. Future studies should compare the predictive ability of this model with frailty indexes from electronic healthcare data. Investigating specific components of CGA and their contribution to health outcomes will also be important.

## Data Availability

The datasets used and/or analysed during the current study are available from the corresponding author on reasonable request.
